# Molecular Characterization of *Escherichia coli* Producing Extended-Spectrum ß-Lactamase and MCR-1 from Sick Pigs in a Greek Slaughterhouse

**DOI:** 10.3390/antibiotics12111625

**Published:** 2023-11-14

**Authors:** Ermioni Avgere, Christos Zafeiridis, Kassandra A. Procter, Apostolos Beloukas, Panagiota Giakkoupi

**Affiliations:** 1 Department of Biomedical Sciences, University of West Attica, 12243 Athens, Greece; ermioneaugere@gmail.com (E.A.); kprokter@uniwa.gr (K.A.P.); abeloukas@uniwa.gr (A.B.); 2 Public Health Policy Department, University of West Attica, 11521 Athens, Greece; chzafeiridis@uniwa.gr; 3 Ministry of Rural Development and Food of Greece (General Directorate of Veterinary Services), Seconded National Expert to the European Commission (Directorate General of Health and Food Safety-Unit G4, Official Controls-Northern Ireland Liaison Team), Belfast BT96DR, UK; 4 National AIDS Reference Centre of Southern Greece, Department of Public Health Policy, University of West Attica, 11521 Athens, Greece; 5 Laboratory for the Surveillance of Infectious Diseases-LSID, Department of Public Health Policy, University of West Attica, 11521 Athens, Greece

**Keywords:** ESBL, pigs, plasmid typing, MLST

## Abstract

The first prospective surveillance of ESBL and colistin-resistant *Escherichia coli* recovered from sick pigs from a slaughterhouse in Central Greece aimed to investigate the spread of relevant genetic elements. In February 2021, 25 *E. coli* isolates were subjected to antimicrobial susceptibility testing using disk diffusion and broth microdilution techniques. PCR screening was conducted to identify ESBLs and *mcr* genes. Additional assays, encompassing mating-out procedures, molecular typing utilizing Pulsed-Field Gel Electrophoresis, multilocus sequence typing analysis, and plasmid typing, were also conducted. A 40% prevalence of ESBLs and an 80% prevalence of MCR-1 were identified, with a co-occurrence rate of 32%. The predominant ESBL identified was CTX-M-3, followed by SHV-12. Resistance to colistin, chloramphenicol, cotrimoxazol, and ciprofloxacin was detected in twenty (80%), fifteen (60%), twelve (48%), and four (16%) isolates, respectively. All *bla*_CTX-M-3_ harboring plasmids were conjugative, belonging to the incompatibility group IncI1, and approximately 50 kb in size. Those carrying *bla*_SHV-12_ were also conjugative, classified into incompatibility group IncI2, and approximately 70 kb in size. The mcr-1 genes were predominantly located on conjugative plasmids associated with the IncX4 incompatibility group. Molecular typing of the ten concurrent ESBL and MCR-1 producers revealed seven multilocus sequence types. The heterogeneous population of *E. coli* isolates carrying resistant genes on constant plasmids implies that the dissemination of resistance genes is likely facilitated by horizontal plasmid transfer.

## 1. Introduction

Acquired antibiotic resistance has emerged as a global concern in both the medical and husbandry sectors since the turn of the century [1]. Consequently, conventional treatments for human and animal infections are increasingly reliant on newer antibiotics, which pose economic challenges for patients and healthcare systems and, in many countries, face accessibility issues [1]. Studying antibiotic resistance is no longer confined to healthcare facilities, as nearly all ecosystems contribute to the emergence, acquisition, and dissemination of antimicrobial-resistant genes. Effectively addressing the surge in antibiotic resistance necessitates embracing the One Health concept [2], integrating knowledge across biological elements crucial for understanding the evolution of antimicrobial resistance. This includes microorganisms or vectors involved in emergence and dissemination, host organisms, whether humans or animals, and their associated habitats [3].

Third-generation cephalosporins, vital for treating human infections caused by Enterobacterales, are concurrently used in hospital settings and for bacterial infections in food-producing animals. The World Health Organization (WHO) and the World Organisation for Animal Health (OIE) classify current antibiotics as clinically important [4,5], with international guidelines emphasizing prudent use. Concerns about the selection and spread of resistance from animals to humans, arising from mass administration in large animal populations for therapy or prophylaxis, underscore the need for careful antibiotic management. Resistance to broad-spectrum cephalosporins and aztreonam in Enterobacterales primarily results from the acquisition of Extended Spectrum ß -Lactamase genes, particularly *bla*_CTX-M_-like and *bla*_SHV_-like genes [3].

The widespread prevalence of ESBLs and carbapenemases in Enterobacterales derived from infections associated with human and animal health settings has forced clinicians to resort to older antibiotics like colistin, despite confirmed side effects [6]. Colistin’s dual use in livestock feed as a growth supplement and as an antimicrobial agent in veterinary medicine in certain countries raises further concerns [6]. Polymyxins, including colistin, are classified as critically important antibiotics for human health by the WHO [4] and as highly important antibiotics for veterinary use by the OIE [5], emphasizing the need for international guidelines. Until 2016, polymyxin resistance was reported to be chromosomally mediated, typically related to mutations in genes regulating the two-component system for lipid A biosynthesis and charge regulation in lipopolysaccharide (LPS) [7,8]. However, the discovery of the plasmid-borne colistin resistance gene mcr-1 in animals and humans in China in 2016 [9], along with the rapid emergence of mutant derivatives (mcr-2 to mcr-9) globally, has raised significant concerns about the facile transferability of polymyxin resistance genes [10]. mcr-like genes have been isolated from human, environmental, and animal samples, predominantly in *Escherichia coli*. [6]. Notably, mcr-like genes have been widely identified among *E. coli* strains recovered from pigs on farms globally, spanning Europe and Greece [11,12].

Antimicrobial resistance in food-producing animals has garnered global attention. In 2021, Greece actively participated in “The COST Action Proposal OC-2021-1-25215 European Antimicrobial Resistance Surveillance network in Veterinary medicine” and implemented the National Monitoring Plan for Antimicrobial Resistance in diseased animals, with a specific focus on bovine and swine populations. In collaboration with thirteen EU/EEA (European Union/European Economic Area) countries, a national committee worked to establish the foundational methodology for the European Antimicrobial Resistance Surveillance Network in Veterinary Medicine (EARS-Vet) [13].

Recent surveys revealed a high incidence of ESBL-producing *Escherichia coli* exceeding 88% in the Greek swine industry, along with approximately 10% for MCR-related Enterobacterales [12]. The exchange of MCR- and ESBL-producing *E. coli* isolates between humans and animals occurs through various pathways, including direct contact and the food chain [14]. Moreover, genes may spread between bacterial isolates through the exchange of mobile genetic elements, such as plasmids [3]. Despite these findings, molecular typing surveys to ascertain whether the pork production chain serves as a reservoir and transmission route for multidrug-resistant bacteria are currently lacking.

This study represents the first molecular epidemiological surveillance of ESBL and MCR producers in *Escherichia coli* from pigs in Greece. In accordance with the requirements of the European program [13], sampling was performed on diseased animals. With a specific focus on prevalent ESBL and mcr genes, particularly within the Greek swine industry, our research aims to identify potential reservoirs and transmission routes of multidrug-resistant bacteria within the pork production chain.

## 2. Results

### 2.1. Resistance/Sensitivity Testing

All 25 (100%) *E. coli* isolates exhibited resistance to penicillins but showed susceptibility to penicillin/inhibitors combinations, cefoxitin, meropenem, and aminoglycosides. Additionally, colistin MIC values for the entire set of 25 isolates fell within the resistant range of 4–32 mg/L, with MIC_50_ at 8 mg/L (Table 1). Ten (40%) *E. coli* isolates were found to be resistant to 3rd generation cephalosporins. Only one isolate (4%) exhibited simultaneous resistance to 3rd generation cephalosporins, ciprofloxacin, cotrimoxazole, and chloramphenicol. Five isolates (20%) were resistant to 3rd generation cephalosporins along with chloramphenicol, and four isolates (16%) were resistant to 3rd generation cephalosporins along with cotrimoxazole (Table 1). Out of fifteen non-ESBL producers, two isolates (13%) demonstrated resistance to chloramphenicol, while five isolates (33%) were concurrently resistant to chloramphenicol and cotrimoxazole. Additionally, three isolates (20%) were resistant to chloramphenicol, cotrimoxazole, and ciprofloxacin (Table 1).

### 2.2. Identification of ESBL Producers

All ten isolates exhibiting resistance to 3rd generation cephalosporins were positive in the DDS test, compatible with ESBL production. The ESBL phenotypes were associated with the presence of *bla*_CTX-M_ genes in eight isolates, specifically *bla*_CTX-M-3_ (99% homology with MH463250) and associated with ISEcp1. Furthermore, two ESBL-positive isolates possessed the *bla*_SHV-12_ ß-lactamase gene (homology 98% to GU299861) (Table 1). No AmpC or TEM producers were detected.

### 2.3. Identification of MCR-1 Producers

Out of the 25 isolates, twenty (80%) were identified as producers of MCR-1, with eight of them (32%) concurrently producing ESBL (Table 1). The production of MCR-1 did not influence the MIC values of colistin. Both MCR-1 producers and nonproducers exhibited MIC values ranging from 4 to 32 mg/L, with the same MIC_50_ values at 8 mg/L. 

### 2.4. Molecular Typing

**Figure 1:**
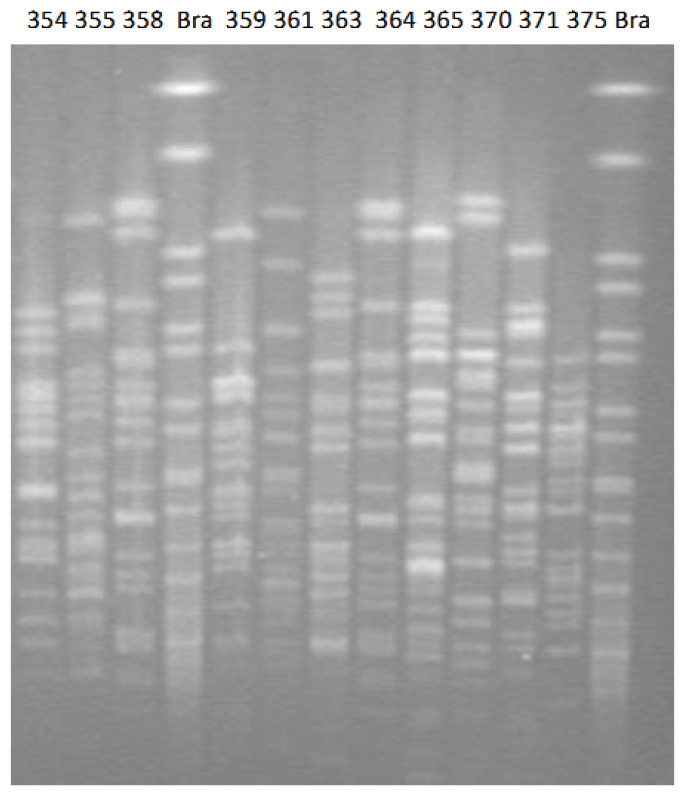
PFGE molecular typing of ESBL producing *E. coli* derived from pigs. *Salmonella* Braenderup H9812 (Bra) was employed as the DNA ladder.

PFGE revealed a diversity of molecular fingerprints (Figure 1). All patterns were considered unrelated to each other, suggesting three or more independent genetic events (seven or more band differences). This implies that all isolates were allocated into different PF types. MLST for the ten ESBL producers identified seven types: ST7, 66, 77, 357, 823, 835, and 1076 (Table 1). ST77 included both SHV-12 producers, while ST7 involved both non-MCR-1 producers. The international high-risk clone ST131 was not detected.

### 2.5. Plasmid Characterization

All eight CTX-M-3-producing isolates demonstrated the transfer of the responsible gene through conjugation, occurring at a frequency of 10^−4^/donor cell. The resulting transconjugants uniformly carried a single plasmid with an approximate size of ~50 kb, exhibiting no additional resistance traits aside from extended-spectrum cephalosporin resistance. These plasmids were consistently allocated to the IncI1 incompatibility group (Table 1).

In the case of SHV-12 producers, both transferred the *bla*_SHV-12_ gene through conjugation at a frequency of 10^−6^/donor cell. The transconjugants harbored a single plasmid sized ~70 kb, displaying no other resistance traits except for extended-spectrum cephalosporin resistance. The plasmids harboring *bla*_SHV-12_ plasmids consistently belonged to the incompatibility group IncI2 (Table 1).

For MCR-1 producers, the conjugative plasmids they carried were predominantly allocated to the IncX4 incompatibility group. Conjugation frequency did not surpass 10^−5^/donor cell (Table 1).

## 3. Discussion

The present study is the first, to the best of our knowledge, to describe the molecular characteristics of *E. coli*-producing ESBL and MCR-1 isolates, sourced from a pig farm in Central Greece. Geographically, central and western Greece host the highest pig density, constituting more than 50% of the total pig population of Greece [12]. 

Antibiotic sensitivity-resistance profiles from our investigation aligned with the MIC values for 3rd generation cephalosporins and cotrimoxazole, mirroring the recent findings of Tsekouras (2022) [12], who examined healthy pigs in various regions of Greece. Notably, Tsekouras reported ESBL-producing *E. coli* with fluoroquinolone resistance exceeding 50% and aminoglycoside resistance around 30%, diverging from our observations but consistent with Michael’s study (2017) in German husbandry [15]. The disparity may be attributed to our study’s limited sample size. 

CTX-M-3 Extended Spectrum ß-lactamase emerged as the predominant ESBL, identified in eight of the 25 isolates. The corresponding gene was housed within a single conjugative plasmid assigned to the IncI1 incompatibility group. The first documented instance of CTX-M-3 in Greece dates back to 1999, when it was isolated from a blood culture in a Peiraeus tertiary hospital [16]. Thirteen years later, it reappeared in a ciprofloxacin-resistant collection of human-derived *Escherichia coli* from Central Greece [17]. In contrast to our findings, CTX-M-3 has not been reported in husbandry in either the national collection by Tsekouras [12] or in the European collection by Ewers [11], both sourced from healthy animals. Michael’s earlier collection (2008–2014) included a minimal fraction (1%) of CTX-M-3-producing *E. coli* from diseased bovine [15]. 

On the other hand, the production of SHV-12 was consistent across all *E. coli* collections that originated from swine [11,12,15]. Within our study, the *bla*_SHV-12_ gene was detected in two out of 25 isolates, residing on a single conjugative plasmid assigned to the IncI2 incompatibility group. Tsekouras’ national collection reported a 4.5% incidence of SHV-type ESBL [12], while Ewers’ European collection highlighted SHV-12 as the predominant variant in swine-derived *E. coli*, reaching 20% [11]. The corresponding gene in Ewers’ study was harbored by a plasmid affiliated with the IncI1 incompatibility group, whereas IncI2 was found in less than 10 instances across all animal specimens in the current collection [11]. 

Furthermore, in our samples obtained from pigs suffering from diarrhea and treated with colistin, all isolated *E. coli* strains exhibited resistance to colistin. Despite an 80% incidence of MCR-1, colistin resistance was likely a consequence of chromosomal mutations in the *pmrB* gene within the two-component regulatory system for the biosynthesis of lipid A. It was observed that there was no discernible difference in MIC values between MCR-1 producers and nonproducers. Additionally, exposure of Enterobacterales to colistin, both in vivo and in vitro, has been reported to induce the emergence of chromosomal colistin resistance in these strains [18,19]. However, chromosomal mutations were not explored in the current study. In our study, the *mcr-1* gene was located on a conjugative plasmid affiliated with the X4 incompatibility group. Tsekouras’ national collection reported low frequencies of colistin-resistant plasmid genes mcr-1, -4, and -8 [12]. In Ewers’ European collection, no *mcr* gene was detected in pigs [11], and the mcr-1 gene’s location within the X4 plasmid incompatibility group was noted in bovine and poultry specimens.

Adhering to OIE guidelines [5], the Greek Ministry of Rural Development and Food has imposed restrictions on the use of 3rd generation cephalosporins and colistin in food-producing animals. According to the “Antibiotics prudent responsible use” program, both 3rd generation cephalosporins and colistin are categorized in group B, to be used only when no alternative exists (Greek Ministry of Rural Development and Food-https://www.minagric.gr/images/stories/docs/agrotis/ktiniatrika_Farmaka/EMA_antiviotika_zoa190620.pdf, accessed on 14 October 2023). As a consequence, a constrained use of both antibiotics is anticipated, leading to a reduction in antibiotic selection pressure. The identification of the gene *bla*_CTX-M-3_ on a conjugative plasmid, which is allocated into incompatibility group I1, aligns with Fischer’s (2014) discovery [20] that IncI1-carrying plasmids impose no or minimal fitness costs on their *E. coli* host and possess the ability to persist even in the absence of antimicrobial selection pressure.

Within the current slaughterhouse, among ESBL producers, seven distinct *E. coli* Sequence Types (STs) were identified, with ST7 being the sole type previously associated with human infections [21]. The remaining STs, such as ST77 and ST823, are exclusively correlated with animal sources [22]. 

Molecular features determined for *E. coli* and their originating plasmids from Greek pigs have predominantly been linked to husbandry-derived isolates [11,15,22]. Concerning the potential exchange of resistance between pigs and humans, our preliminary results support that pigs do not act as direct reservoirs in the transmission of Extended Spectrum ß-Lactamase (ESBL) genes to *E. coli* in humans. This is substantiated by the absence of human-associated bacterial types detected in the *E. coli* isolates derived from pigs. Furthermore, a comprehensive examination of ESBL and mcr-1 transconjugants revealed that the corresponding plasmids did not carry any other resistant traits. *E. coli* isolates exhibiting different Multi Locus Sequence (MLS) types and Pulsed-Field (PF) fingerprints, characterized as unrelated isolates, harbored ESBL gene-carrying plasmids with similar sizes and lacked any other resistant genes. This points toward the horizontal dissemination of these plasmids.

In conclusions, this study stands as a pioneer in elucidating the molecular characteristics of ESBL and MCR-1 producing *E. coli* isolated from a pig farm in Central Greece. While our study provides crucial molecular insights into antibiotic resistance in Greek pig farming, it acknowledges limitations, including a relatively small sample size, a single day of sampling, and a focus on diseased animals in a specific region. To achieve a comprehensive understanding of antibiotic-resistant microbial transmission within the One Health framework, more extensive molecular typing surveys are imperative, both in Greece and globally. A more detailed survey of the presence of certain antibiotic-resistant genes on specific plasmids and bacterial strains is warranted. The strength of this study lies in its pioneering focus on molecular characteristics, shedding light on the dynamic interplay of resistant genes, plasmids, and bacterial strains in the Greek pig population. These findings contribute to the broader discussion on antibiotic resistance dynamics and highlight the need for targeted interventions and surveillance strategies in livestock settings.

## 4. Materials and Methods

### 4.1. Bacterial Isolation and Antimicrobial Susceptibility Testing

A total of 25 pig loose stool samples were collected from weaned piglets with coliform bacterial diarrhea, treated with colistin, in a Central Greece slaughterhouse on a single day in February 2021. Sampling was performed on account of “The COST Action Proposal OC-2021-1-25215 European Antimicrobial Resistance Surveillance network in Veterinary medicine” [13], which collected specimens from sick animals, and all permissions required have been accomplished. 

Twenty-five *Escherichia coli* isolates were recovered from stool cultures on TBX selective chromogenic agar (ThermoFisher, Scientific Inc., Waltham, MA, USA) and subjected to resistance/susceptibility testing for ß-lactams, aminoglycosides, cotrimoxazole, chloramphenicol, and ciprofloxacin following EUCAST instructions. Minimum inhibitory concentrations (MICs) of colistin were determined using broth microdilution in cation-adjusted Mueller-Hinton broth MICRONAUT MIC–Strip Colistin–MERLIN (Diagnostika GmbH, Bornheim–Germany), and results were interpreted according to the EUCAST/CLSI joint guidelines. Phenotypic ESBL detection was performed by the Double Disk Synergy Test (DDST) following EUCAST instructions [23].

### 4.2. Genotypic Characterization of Resistance Determinants

DNA purification was performed using Nucleospin Tissue, Macherey Nagel GmbH & Co. KG (Düren, Germany). Molecular identification of ESBL-encoding genes was performed by PCR using previously published primers [24] and subsequent sequencing. PCR amplicons were subjected to Sanger sequence analysis (CeMIA SA, http://cemia.eu/sangersequencing.html, accessed on 14 October 2023), as previously described [25]. PCR screening for plasmid-mediated colistin resistance genes (mcr-1 to -5) was performed following EUCAST guidelines for detection of resistance mechanisms and specific resistances of clinical and/or epidemiological/importance (Version 2.01, July 2017) [23].

### 4.3. Mating-Out Assays and Plasmid Analysis

A liquid-mating assay with *E. coli* 1R716, as a recipient, investigated the transferability of resistant determinants, as described previously [24]. Transconjugants were selected on LB agar supplemented with streptomycin (2000 mg/L), ceftriaxone (2 mg/L), and ceftazidime (1 mg/L) for ESBL-producing isolates and colistin (2 mg/L) for mcr-1-positive isolates. Transconjugants were subjected to PCR for confirmation and antimicrobial susceptibility testing. The presence of a unique plasmid in each transconjugant was confirmed by digestion with S1 nuclease (ThermoFisher, Scientific Inc.). This endonuclease introduces single-stranded nicks and breaks in duplex DNA to convert a circular plasmid to a linear one [26]. The size of each plasmid was calculated by subsequent PFG electrophoresis [26]. Plasmid DNA was purified by Nucleospin plasmid MIDI, Macherey Nagel GmbH & Co.KG. The PCR-based replicon typing (PBRT) method was carried out on all transconjugants, as previously described [27].

### 4.4. Clonality Evaluation

Pulsed-field gel electrophoresis (PFGE) assessed the clonal relationship of *E. coli* isolates. Total DNA from *E. coli* isolates was digested using the *Xba*I enzyme (New England BioLabs, Ipswich, MA, USA). Then, the generated fragments were separated by PFGE using a CHEF-DR III System (Bio-Rad, Cressier, Switzerland) [25]. A comparison of molecular fingerprints was performed visually based on Tenover’s 1995 publication [28]. Multilocus sequence typing (MLST) according to the Pasteur scheme was performed for ESBL-producing isolates.

## Figures and Tables

**Table 1:** Features of *E. coli* derived from pigs and their plasmids harboring ESBL and mcr-1 resistant genes.

No	Isolation Date	ESBL	AmpC	MCR-1	MLST	ESBL Plasmid			Mcr-1 Plasmid			MIC Col (mg/L)	Resistance to Antibiotics Other than β-Lactams and Colistin
						Conjugation Frequency	PBRT	S1 Based Size	Conjugation Frequency	PBRT	S1 Based Size		
A358	6/2/2021	CTX-M-3	-	+	1076	10^−4^	IncI1	~50 kb	10^−5^	IncX4	ND	32	
A359	6/2/2021	CTX-M-3	-	+	367	10^−4^	IncI1	~50 kb	10^−5^	IncX4	ND	16	sxt, cm, cip
A363	6/2/2021	CTX-M-3	-	+	835	10^−4^	IncI1	~50 kb	10^−5^	IncX4	ND	16	
A364	6/2/2021	CTX-M-3	-	+	1076	10^−4^	IncI1	~50 kb	10^−5^	IncX4	ND	16	
A365	6/2/2021	CTX-M-3	-	+	823	10^−4^	IncI1	~50 kb	10^−5^	IncX4	ND	4	
A375	6/2/2021	CTX-M-3	-	+	66	10^−4^	IncI1	~50 kb	10^−5^	IncX4	ND	8	
A370	6/2/2021	CTX-M-3	-	-	7	10^−4^	IncI1	~50 kb	-	-	-	4	cm
A371	6/2/2021	CTX-M-3	-	-	7	10^−4^	IncI1	~50 kb	-	-	-	16	sxt, cm
A354	6/2/2021	SHV-12	-	+	77	10^−6^	IncI2	~70 kb	10^−5^	IncX4	ND	16	sxt, cm
A361	6/2/2021	SHV-12	-	+	77	10^−6^	IncI2	~70 kb	10^−5^	IncX4	ND	8	sxt, cm
A355	6/2/2021	-	-	+	ND	-	-	-	-	-	-	8	
A356	6/2/2021	-	-	-	ND	-	-	-	-	-	-	32	cm
A357	6/2/2021	-	-	+	ND	-	-	-	-	-	-	4	sxt, cm, cip
A360	6/2/2021	-	-	+	ND	-	-	-	-	-	-	8	
A362	6/2/2021	-	-	+	ND	-	-	-	-	-	-	4	cm
A366	6/2/2021	-	-	+	ND	-	-	-	-	-	-	8	
A367	6/2/2021	-	-	+	ND	-	-	-	-	-	-	4	
A368	6/2/2021	-	-	+	ND	-	-	-	-	-	-	16	sxt, cm
A369	6/2/2021	-	-	+	ND	-	-	-	-	-	-	16	sxt, cm
A372	6/2/2021	-	-	-	ND	-	-	-	-	-	-	8	sxt, cm
A373	6/2/2021	-	-	+	ND	-	-	-	-	-	-	16	sxt, cm, cip
A374	6/2/2021	-	-	+	ND	-	-	-	-	-	-	32	
A376	6/2/2021	-	-	+	ND	-	-	-	-	-	-	32	sxt, cm
A377	6/2/2021	-	-	-	ND	-	-	-	-	-	-	4	sxt, cm, cip
A378	6/2/2021	-	-	+	ND	-	-	-	-	-	-	8	sxt, cm

ND: not determined; col: colistin; sxt: cotrimoxasol; cm: chloramphenicol; cip: ciprofloxacin.

## Data Availability

Data are contained within the article.
